# Traditional Chinese medicine for anti-Alzheimer’s disease: berberine and evodiamine from *Evodia rutaecarpa*

**DOI:** 10.1186/s13020-020-00359-1

**Published:** 2020-08-05

**Authors:** Zhiling Fang, Yuqing Tang, Jiaming Ying, Chunlan Tang, Qinwen Wang

**Affiliations:** Department of Preventative Medicine, Zhejiang Provincial Key Laboratory of Pathological and Physiological Technology, Medical School of Ningbo University, Ningbo, 315211 Zhejiang China

**Keywords:** Alzheimer’s disease, Pathogenic hypothesis, Berberine, Evodiamine, Traditional Chinese medicine

## Abstract

Alzheimer’s disease (AD) is one of the most common diseases in elderly people with a high incidence of dementia at approximately 60–80%. The pathogenesis of AD was quite complicated and currently there is no unified conclusion in the academic community, so no efficiently clinical treatment is available. In recent years, with the development of traditional Chinese medicine (TCM), researchers have proposed the idea of relying on TCM to prevent and treat AD based on the characteristic of multiple targets of TCM. This study reviewed the pathological hypothesis of AD and the potential biomarkers found in the current researches. And the potential targets of berberine and evodiamine from *Evodia rutaecarpa* in AD were summarized and further analyzed. A compound-targets-pathway network was carried out to clarify the mechanism of action of berberine and evodiamine for AD. Furthermore, the limitations of current researches on the TCM and AD were discussed. It is hoped that this review will provide some references for development of TCM in the prevention and treatment of AD.

## Background

Alzheimer’s disease (AD) is a common neurodegenerative disease with a high incidence of dementia (60–80%), which has been listed as the sixth leading cause of death in the United States by the Centers for Disease Control and Prevention. It is estimated that, by the middle of this century, the number of Alzheimer’s patients in the United States will reach 5.4 million, and the total number is expected to reach 15 million [1]. Currently, one person develops into AD every 66 s [2], 44 million people worldwide have AD or related dementia. Unfortunately, this ratio is expected to triple by 2050 [3]. The main symptoms of AD include memory loss, cognitive impairment, and loss of self-care eventually. Therefore, AD has become the main form of special care units in nursing homes [4]. The caring for AD patients is not only a financial burden, but also a psychological one for the entire family. Considering this situation, the prevention and treatment of AD is a huge challenge to public health and medical systems, making it more important to strengthen basic research of AD.

In the past 30 years, a variety of resources have been invested to develop drugs for AD. Currently, there are only clinical drugs approved by Food and Drug Administration (FDA) for treatment of AD. Four of them are acetylcholinesterase inhibitors, including tacrine, donepezil, rivastigmine and galantamine [5]. These drugs demonstrated certain therapeutic effects but the side effects, including nausea, diarrhea, insomnia and a slower heart rate, cannot be ignored. Another drug is memantine, which blocks the neurotransmitter glutamate. These drugs can potentially delay the progression of memory loss but cannot completely cure AD patients, because AD is a quite complex disease that may not be cured based on one target.

Traditional Chinese medicine (TCM) has been used for the prevention and treatment of neurodegenerative disease in China and other Asian countries for more than 3000 years [6]. The holistic view is the core concept of TCM. It is usually used in the form of multi-target and multi-channel treatment, which satisfy the prevention and treatment of multi-target and complex diseases. In recent years, many researchers have found that many herbs and ingredients isolated from herbs have good efficacies to AD with fewer side effects. Ginkgo biloba extract, such as EGB761, can improve cognitive function, neuropsychiatric symptoms and functional abilities in AD [7]. Monomers such as baicalein [8], tanshinone [9] and huperzine A [10] extracted from herbal medicines are also proved to demonstrate curative effects against AD. Therefore, TCMs are expected to become promising candidates for the prevention and treatment of AD.

In this study, the pathogenesis of AD and potential biomarkers of AD found in current researches were reviewed. And the targets of berberine and evodiamine from *Evodia rutaecarpa* were summarized and further analyzed. A compound-targets-pathway network was carried out to clarify the mechanism of action of berberine and evodiamine. Furthermore, the limitations of current researches on TCM and AD were discussed, which might promote the development of effective disease-modifying TCM monomers or extracts.

## Pathological hypothesis of AD

The Aβ hypothesis and tau protein hypothesis are the two most accepted hypotheses for AD. Aβ or β-amyloid protein is the product of the sequential cleavage of amyloid precursor proteins by β-secretase and γ-secretase. This type of cutting produces other molecules that may also play a role in AD progression, but Aβ is the most important one. This protein is prone to fold incorrectly and aggregate into oligomers, which deposit in the brain to form plaques eventually and affect the normal function of brain. Current technologies cannot detect which types of oligomers are toxic. Extracellular Aβ aggregates into neurotic plaques, which now is attributed to cerebral amyloid vascular disease. In the early stage of disease, diffused Aβ plaques could be observed in the frontal and parietal lobes. With the progression of AD, diffused plaques and neurogenic plaques will be further discovered in the broader neocortical region, typically in the following order of spread: neocortex, hippocampus, basal ganglia, brainstem, and cerebellum [11]. The impact of Aβ is also regulated by tau protein. The tau protein is usually highly phosphorylated and abnormally shaped in the brain of AD patients. The insoluble tau protein also aggregates into various forms. Additionally, the pathological tau protein can affect healthy neurons nearby, causing the misfolding of protein to spread throughout the whole brain. The proliferation of tau accumulation is often referred to as prion-like proteins, demonstrating the ability to induce the same abnormal conformation in homologous proteins, triggering a self-amplification cascade eventually [12]. Several studies have shown that there is a causal relationship between Aβ and formation of P-Tau (Phosphorylated-Tau). In fact, tau as an axonal protein, has a dendritic function in postsynaptic targeting of the Src kinase Fyn, a substrate of which is the *N*-methyl-d-aspartic acid (NMDA) receptor. Animal experiments have shown that this dendritic effect of tau contributes to post-synaptic toxicity of Aβ and reducing endogenous tau levels prevents behavioral deficits in transgenic mice expressing human amyloid precursor protein [13, 14]. In addition, Talantova et al. [14] found that oligomeric Aβ caused glutamate release from astrocytes, and then activated synaptic NMDA receptor, leading to increased levels of P-Tau. All of these prove the toxicity of Aβ depends on tau. This regulation is not about overexpressing or reducing content of Aβ but matters the neurotoxicity. Yet the increase of P-Tau expression is associated with the total reduction in the number of synaptic [15]. In fact, the basis of a series of neurodegenerative diseases is exactly the pathological accumulation of P-Tau related to microtubules in nerve cells and glial cells. Abnormally highly P-Tau cannot bind to microtubules like normal proteins. Tau, which comprises a filamentous bundle of neuronal fibers, is inert and fails to stimulate microtubule assembly, causing microtubules break ultimately [16].

Inflammation in neurons is also considered an important factor in the development of AD. Microglia and astrocytes are two major glial cell types in the pathogenesis of AD [17]. Microglia cells are immune effector cells of central nervous system (CNS) and play an important role in the immune response. Astrocytes are the most abundant glial cells in the CNS. They can regulate the pH value, ion homeostasis, oxidative stress and blood flow, perform fine control of the environment, and provide nutritional and metabolic support for neurons [18, 19]. Glial cells are highly heterogeneous, and their activation can protect the brain by responding quickly to brain injury. However, uncontrolled and prolonged activation would have the opposite effect. In this case, microglia acquire a pro-inflammatory phenotype. The release of pro-inflammatory molecules, reactive oxygen species (ROS), and nitric oxide will cause neuronal death. The pathways of the tumor necrosis factor (TNF, inflammatory factor) have been well studied. TNF usually circulates in the blood, enters the ventricle by passing through the blood-brain barrier (BBB), and affects local cells through tumor necrosis factor receptor, causing activation of the c-Jun N-terminal kinase pathway and nuclear factor κ-light-chain-enhancer of activated B-cell (NFκB) cascade and leading to increased TNF production [20]. Additionally, TNF can increase glycogen synthase kinase 3β (GSK-3β) by acting on phosphoinositide 3-kinases (PI3K) and mitogen-activated protein kinases (MAPK), leading to amyloid deposition. Other studies have also demonstrated that TNF-α can increase the phosphorylation of active protein 1 as a catalyst, an activity that is directly related to P-Tau [21, 22]. Additionally, amyloid β can activate NADPH oxidase to enhance the production of ROS in astrocytes, leading to mitochondrial dysfunction and astrocytes consumption [23]. Many researchers believe that this interaction between the CNS inflammatory response and amyloid protein is the core of AD [24].

**Fig. 1:**
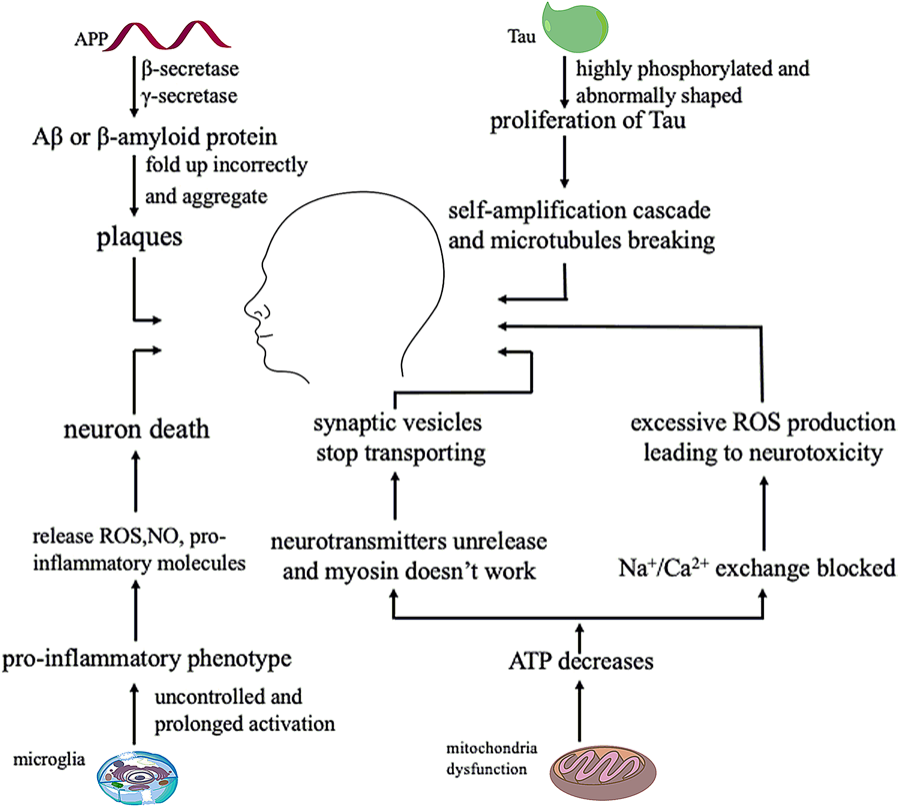
The main pathogenesis of Alzheimer’s disease

Mitochondria are very important organelles for cell production. Increasing evidence on the postmortem brain of AD patients and some laboratory models has proven that mitochondrial dysfunction is the key to the onset of AD, and this event usually occurs in the early stage of AD [25, 26]. In addition to their role in energy metabolism, mitochondria have many key functions, including maintaining proper regulation of intracellular calcium homeostasis, intracellular REDOX balance, and mediating apoptosis and necrosis [27]. First, as a production site, when mitochondria cannot work normally, the amount of ATP decreases and the synaptic structure will be the first to be affected. In the neuron, the synaptic structure is the key to maintaining functional neurotransmission. Without ATP-dependent vesicle neurotransmitters to release, or with a lack of ATP for myosin work needed, synaptic vesicles cannot normally transport [28]. A synthetic defect in a neurotransmitter called acetylcholine (ACh) is thought to underlie cognitive impairment [29]. In fact, at the molecular level, the cholinergic hypothesis is the first and most studied way to describe the pathophysiology of AD. This selective change leads to the downregulation of cholinergic markers, such as acetylcholinesterase (AChE). There is a proportional relationship between changes in cholinergic markers, the density of altered nerve fibers, and the severity of pathology [30]. Notably, a decreased synaptic density, impaired synaptic transmission, and synaptic plasticity defects are typical synaptic pathologies associated with AD [27]. Second, mitochondria buffer Ca^2+^ concentrations and the maintenance of calcium homeostasis are very important at the cellular level. The lack of ATP can reduce the available energy for the Na^+^/Ca^2+^ exchange process, which is very vital for the removal of Ca^2+^ in neurons, otherwise excessive ROS production may lead to neurotoxicity [31]. Third, mitochondria are significant sites for oxidative phosphorylation. Mitochondrial OXPHOS deficiency and ATP deficiency are the signature pathological features of the AD brain [32]. The phenomenon of reduced activity of mitochondrial complex IV is detected in the platelets of AD patients. [33]. Additionally, Fang’s study demonstrated, for the first time, that PTEN-induced decreased expression of putative kinase 1 (PINK1) was associated with the pathology of AD. Gene therapy-mediated PINK1 overexpression enhances the autophagy signal by activating autophagy receptor (OPTN NDP52), achieving the goal of clearing damaged mitochondria finally and alleviating the synaptic loss caused by amyloid protein and the cognitive decline of AD mice [34]. Recent experiments have shown that the autophagy vacuole contains Aβ protein and the secretase required to produce Aβ, which is especially rich in γ-secretase activity and γ-secretase complex constituent [35]. Although the relationship between mitochondria, cell autophagy and AD still needs further study, the findings have proved the role of mitochondria as the regulating factor in the cell death pathways. The above pathogenesis of AD shows in Fig. 1.

## Biomarkers of AD

The biomarkers of AD have been widely reported. Due to the closeness to the brain and abundant cerebral-specific proteins, cerebrospinal fluid (CSF) is a key biological fluid for deciphering changes in protein levels and exploring the pathways in CNS diseases [36]. It has been widely accepted that amyloid deposition and tau protein in the brain are the most commonly used evaluation indicators in CSF or molecular imaging [37]. Some classic biomarkers, including Aβ42, Aβ40, Aβ38, T-Tau and P-Tau, can be detected in CSF. In the research framework of the National Institute on Aging and Alzheimer’s Association, biomarkers are divided into amyloid deposition (A), pathology tau (T), and neurodegenerative changes (N). The ATN classification system set groups different biomarkers (imaging and biofluids) according to the pathological process of each measurement [38]. Catherine et al. suggested that the best time to introduce interventions may be when amyloid biomarkers are abnormal but not before both tau protein and neurodegeneration are abnormal [39]. Ilijana et al. found an association between strong protein abundance and disease severity [40]. The proteins APLP1 and SPP1 showed the best diagnostic potential in early differentiation between AD and the control group, and the proteins APLP1, SPP1 and CNTN2 may be indicators of disease progression. APLP1, CNTN2 and SPP1 proteins showed a significant correlation with the MMSE and CDR test (*p *< 0.05). Liang Feng et al. compared the plasma levels of 4 lncRNAs between AD patients and non-AD patients and found that the β-amyloid precursor protein lyase 1 (BACE1) level in the plasma of AD patients was increased and was highly specific to AD (88%), suggesting that BACE1 may be a potential candidate biomarker to predict AD [3]. In some experiments, the blood lipid assay was used to determine whether lipid biomarkers could distinguish AD cases from the control group. The results provide additional biomarkers and confirm the possibility of a lipid profile for diagnosis and potential AD staging [41].

Although CSF testing is the most stable and accurate method, it requires lumbar puncture. If necessary, puncture collection from the cerebellar medullary cistern or lateral ventricle is demanded. Because AD patients are mostly elderly, one of the main objectives of neurodegenerative disease research in recent years has been to develop a less-invasive method to evaluate dementia biomarkers. Using other methods to diagnose the different types of dementia will require neuroimaging technology progress, such as positron emission tomography (PET) and magnetic resonance imaging [42]. The different imaging model not only provides complementary information but also the spatial distribution of the measured value can provide abundant information [43]. To some extent, the development of detection technology has broadened the range of biomarkers. The trend to use markers with scanning detection in combination is rising. For example, translocator protein TSPO is an outer mitochondrial membrane protein, which is expressed in many tissues throughout the body [44]. In healthy brains, TSPO is only expressed at low levels and is upregulated in microglia and astrocytes activated and proliferated after brain injury and neuroinflammation [45]. The differential expression of TSPO in activated glial cells allows it to be used in combination with PET [46]. However, TSPO tracers have limitations, including a low binding affinity, high non-specific binding, and a low signal-to-noise ratio [47]. They are affected by the genetic variability of the TSPO binding site, resulting in high-affinity, mixed-affinity, and low-affinity binders [48]. Further research is needed to make it more mature. Additionally, the reduced glucose uptake in specific areas of the brain is associated with AD, whether early-onset AD or late-onset AD; thus, measuring brain glucose metabolism can be used as a metabolic biomarker for the early diagnosis of AD [49]. To track the glycolysis pathway, glucose was labeled with fluorine [50]. Positron emission tomography can be used to measure the absorption of fluorodeoxyglucose in different regions of the brain and can be used for the preliminary diagnosis of AD. The detection of metabolites in urine is also a low-invasive method. For example, the high concentration of neuronal thread protein (NTP) in urine is a typical representative of AD pathology [51]. NTP interacts with antibodies produced against pancreatic thread protein (PTP). Because PTP levels are parallel to the relative concentrations of NTP in cerebrospinal fluid and urine, high NTP levels in urine can be used as a diagnostic tool for AD [52, 53]. The National Institute on Aging 2018 Alzheimer’s Disease Research Summit “road to treatment and prevention” made an important recommendation to carry out precision medical research in the field of AD. The integration of artificial intelligence and neuroimaging data with other omics data will be the key to progress in the field of AD treatment [43]. In addition to biological factors (such as genetics, CSF and blood proteomics) and AD medical risk factors (such as hypertension, diabetes, obesity, and depression), we can also focus on national factors that may be related to the onset, diagnosis time between different ethnic groups, clinical manifestations and differences between AD [54]. Dementia and AD are not easy to diagnose, according to the Alzheimer’s Association, with only 44% of people with dementia being diagnosed in England, Wales and Northern Ireland. Therefore, the innovation and development of methods and techniques for AD diagnosis are very urgent and necessary. The main biomarkers of AD reported in current researches are summarized in Table 1.

**Table 1:** The main biomarkers of Alzheimer’s disease

Biomarker	Change in AD compared with non-AD	Application	References
Aβ42^a^	Decreased in CSF and plasma	Used clinically especially in early stage	[52]
Aβ40	Both Aβ40 alone and the ratio of Aβ42/Aβ40 decreased in CSF and plasma	The ratio of Aβ42/Aβ40 used in research	[53]
Aβ38	Both Aβ38 alone and the ratio of Aβ42/Aβ38 decreased in CSF and plasma	The ratio of Aβ42/Aβ38 used in research	[54]
T-tau^a^	Increased in CSF and plasma	T-tau used in clinic and the ratio of T-tau/Aβ42 used in research	[55]
P-tau^a^	Increased in CSF and plasma	P-tau used in clinic and the ratio of P-tau/Aβ42 used in research	[56]
BACE1	Increased in CSF and plasma	Used widely in research	[57]
hFABP	Increased in CSF	Used widely in research	[58]
TREM2	Increased in CSF TREM2 mRNA increased in blood	Used widely in research	[59]
YKL-40	Increased in CSF	Used widely in research	[60]
SNAP-25	Increased in CSF	Used widely in research	[61]
TDP-43	Increased in CSF and plasma	Used widely in research	[62]
VILIP-1	Increased insignificantly in CSF	Used in disease progression research	[63]
NF-L	Increased in CSF	Used widely in research	[64]
Neurogranin	Increased in CSF	Neurogranin and N/BACE1 ratio used in research	[65, 66]
AD7c-NTP	Increased in urine	Rarely used in research	[49, 50, 67]
24S-OH-Chol	Increased in plasma	Used widely in research	[68]
AAT	Increased in plasma	Used widely in research	[69]
F2-isoprostanes	Increased in CSF	Used in disease progression research	[70]

^a^Represents application in clinic

## TCM for AD

Ehret et al. conducted a systematic literature search of articles published in MEDLINE and EMBASE for the past 10 years to study the treatment status of donepezil, rivastigmine, galantamine and memantine [55]. The results showed that only the effects of cholinergic drugs can show a continuous but insignificant clinical effect. Patients with advanced AD may require higher doses of cholinesterase inhibitors, but this strategy is limited by adverse events, such as nausea, vomiting, and diarrhea [56]. FDA-approved drugs are partial inhibitors rather than therapeutic drugs. These first-line cholinesterase inhibitors are only used to alleviate the symptoms of AD. The effect of a wide range of targeted therapies is more pronounced than the effect of affecting only one target. The “single-molecule-single-target” treatment of AD has largely failed; thus, treatments for the “combination-drugs-multitargets” strategy need to start from multiple perspectives to block the progression of the pathogenesis of AD [57]. TCM has become the focus of attention due to its wide range of pharmacological activities and better protection for patients. Traditionally, some TCMs have been used to treat AD, and they play a crucial role in the discovery of new anti-AD drugs [58]. TCM is a representative of multiple goals and multiple pathways. The ingredients of TCM are complex and diverse, and different components and formulas will act on different targets or pathways. Prevention and health care methods of TCM include mood regulation, seasonal health care, diet health care, herbal medicine, acupuncture, massage, and detoxification. The Chinese government has formulated nearly 100 regulations to support the development of TCM [59]. Some TCMs or its monomers isolated from TCMs have already achieved initial success, including turmeric, tripterygium, ginseng and *E. rutaecarpa* [60–64]. In this study, the two active ingredients of *E. rutaecarpa* were reviewed about their pharmacology and mechanism of action for AD.

## Active ingredients of *Evodia rutaecarpa* for AD

**Fig. 2:**
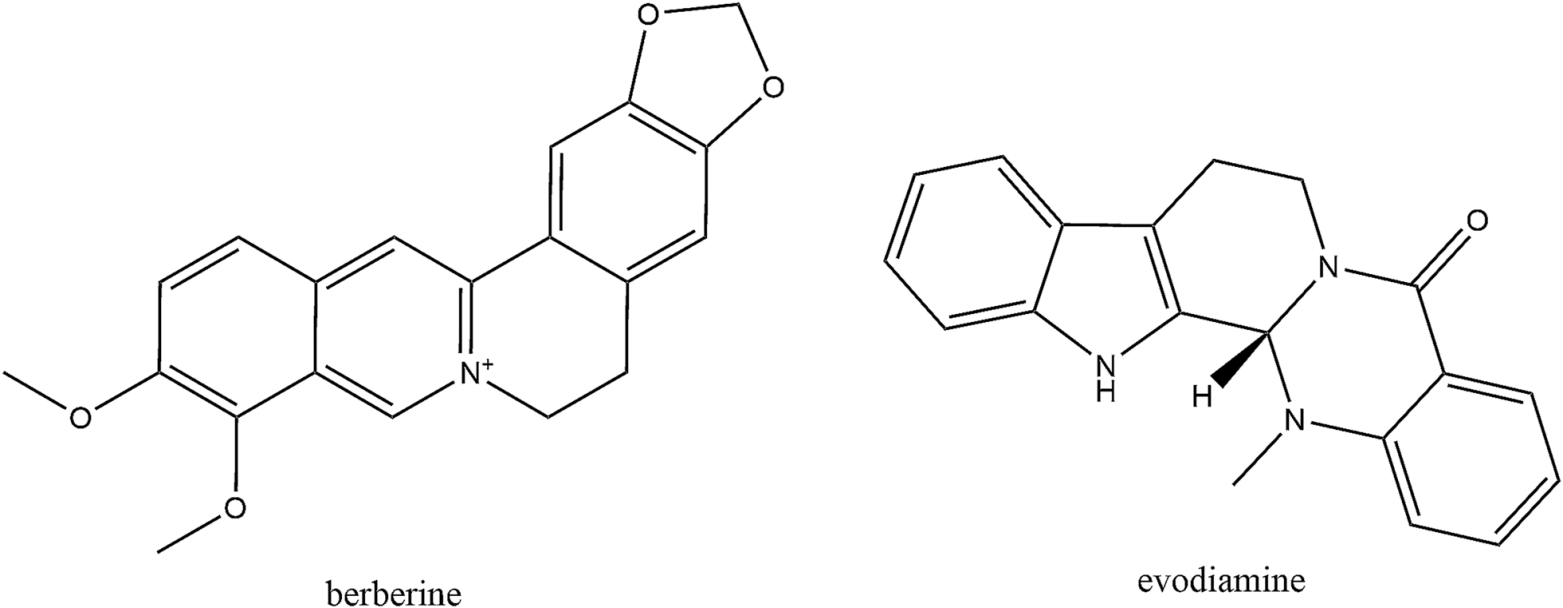
The structures of BBR and Evo

*Evodia rutaecarpa* is a dry, near-mature fruit of the genus *Evodia*, which is used for the treatment of headache, abdominal pain, postpartum hemorrhage, dysentery and amenorrhea [65]. Recently, it was reported to be a potential drug for the control and prevention of AD development and progression [66]. Therefore, screening and summarizing the active ingredients from *E. rutaecarpa* were carried out. The traditional Chinese medicine systems pharmacology database and analysis platform (TCMSP) were used to screen the potential components via oral bioavailability (OB), drug-likeness (DL) and BBB. In general, we believe that substances with an OB value greater than 30% can be well absorbed and metabolized, and the compound has a “drug-like” level of 0.18 as a selection criterion for “drug-like” compounds in TCM [67]. Additionally, in the TCMSP database, the criteria are as follows: compounds with BBB < − 0.3 are considered non-penetrating (BBB−), those with BBB − 0.3 to + 0.3 are considered moderate penetrating (BBB±), and those with BBB > 0.3 are considered strong penetrating (BBB+). Nineteen components of *E. rutaecarpa* with OB greater than 30%, DL greater than 0.18, and BBB greater than 0.3 were selected and are shown in Table 2. Among the 19 components, evodiamine (Evo) and berberine (BBR) have attracted much attention by researchers according to the Pubmed literatures. Therefore, we focused on Evo and BBR in this review and the structures of these two substances are shown in Fig. 2.

**Table 2:** 19 potential activity compounds of *Evodia rutaecarpa*

Number	Molecule name	OB (%)	BBB	DL
1	Evodiamine	86.02	0.85	0.64
2	Goshuyuamide I	83.19	0.64	0.39
3	Evodiamide	73.77	0.81	0.28
4	*N*-(2-methylaminobenzoyl) tryptamine	56.96	0.8	0.26
5	Fordimine	55.11	0.75	0.26
6	Evocarpine	48.66	1.17	0.36
7	1-Methyl-2-[(Z)-undec-6-enyl]-4-quinolone	48.48	1.14	0.27
8	1-Methyl-2-[(Z)-pentadec-10-enyl]-4-quinolone	48.45	1.11	0.46
9	1-Methyl-2-nonyl-4-quinolone	48.42	1.21	0.2
10	1-Methyl-2-undecyl-4-quinolone	47.59	1.19	0.27
11	Icosa-11,14,17-trienoic acid methyl ester	44.81	1.07	0.23
12	1-Methyl-2-pentadecyl-4-quinolone	44.52	1.05	0.46
13	Dihydrorutaecarpine	42.27	0.7	0.6
14	Rutaecarpine	40.3	0.71	0.6
15	24-Methyl-31-norlanost-9(11)-enol	38	1	0.75
16	Beta-sitosterol	36.91	0.99	0.75
17	Sitosterol	36.91	0.87	0.75
18	Berberine	36.86	0.57	0.78
19	1-(5,7,8-Trimethoxy-2,2-dimethylchromen-6-yl) ethanone	30.39	0.75	0.18

*OB* oral bioavailability, *DL* drug-likeness, *BBB* blood–brain barrier

## Comparison of different alkaloids

Alkaloids are very important organic compounds in natural plants, which are synthesized as secondary metabolites in plants and fungi and have extensive biological activities. The presence of at least one nitrogen atom is a general chemical characteristic of it [68]. Among these natural products, alkaloids are considered the most promising candidates for the treatment of AD [69]. According to the molecular model, a binding site of AChE can interact with positively charged nitrogen to inhibit AChE activity [70, 71]. According to the structure, alkaloids can be divided into piperidine alkaloids, isoquinoline alkaloids, indole alkaloids, terpenoids, steroids and other alkaloids, mainly distributed within Buxaceae, Amaryllidaceae and Lycopodiaceae [72, 73]. We compared and summarized the roles of different categories of alkaloids in extraction methods and neurodegenerative diseases, as shown in Table 3.

**Table 3:** Different categories of alkaloids in extraction methods and neurodegenerative diseases

Category	Example	Extraction method	Pharmacological mechanism in AD	References
Piperidine alkaloids	Piperine	Methanol extraction	Reduce GSH, MDA	[74]
Isoquinoline alkaloid	BBR	Water decoction	Inhibits AChE, BChE activity and β-secretase	[75]
Indole alkaloids	Evo	Hot water reflux extraction	Inhibits AChE	[76]
Terpenoids alkaloids	Voacangine	90% ethanol reflux extraction	Inhibits AChE	[77]
Steroidal alkaloids	Conessine	90% ethanol extraction	Inhibits AChE and the activation of NF-κB	[78]
	Corynoxine	70% ethanol reflux extraction	Reduce Ca2+ overload and tau protein hyperphosphorylation	[79]
Other alkaloids	Caffeine	Ethanol extraction	Reduce caspase-3 and β-secretase	[80]
	Isorhynchophylline	70% ethanol reflux extraction	Down-regulate GSK-3β activity and activation of PI3K/Akt signaling pathway	[79, 81, 82]

## Evodiamine

**Fig. 3:**
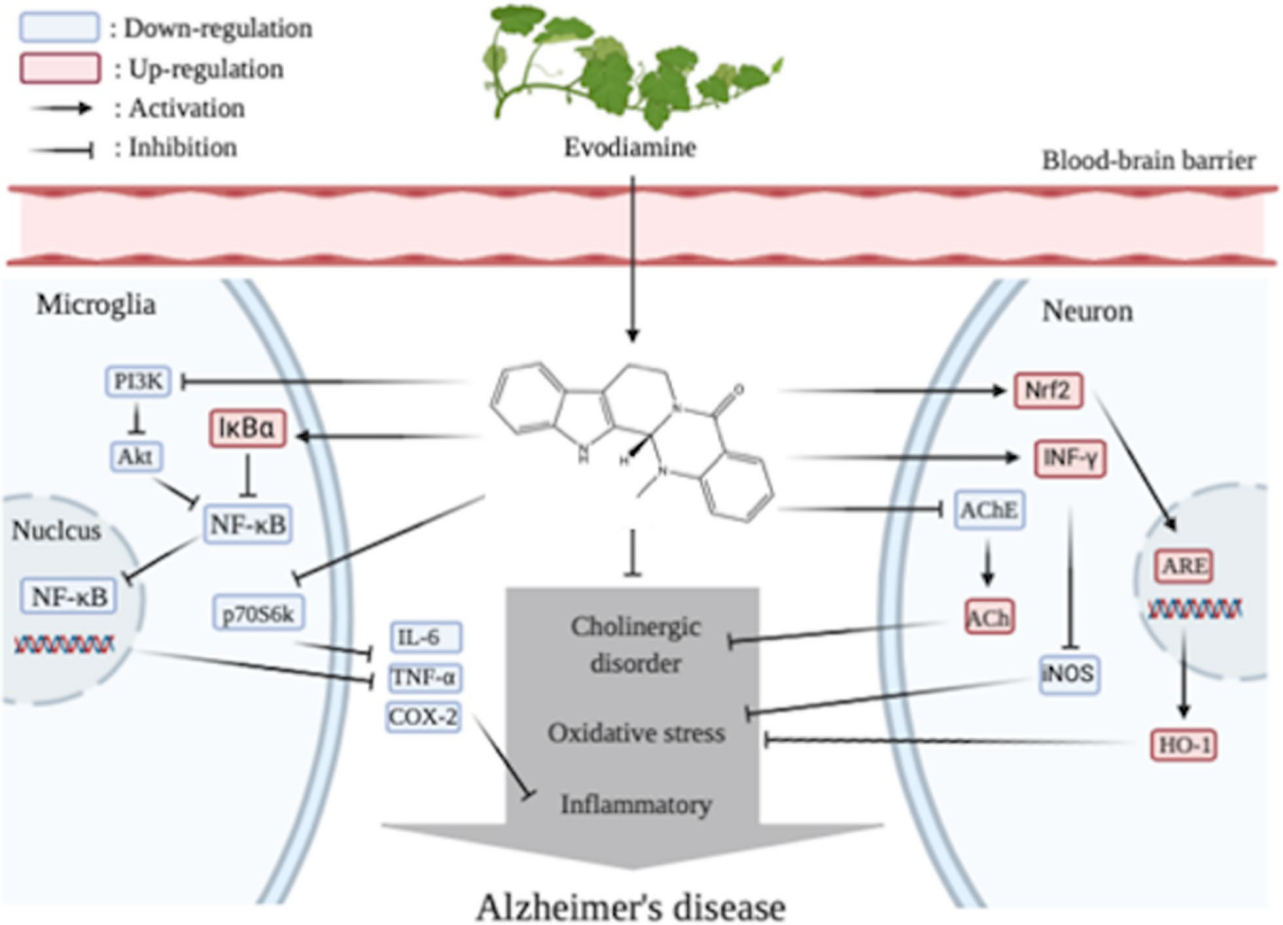
The pharmacodynamic mechanism of Evo for Alzheimer’s disease

Evodiamine (Evo) is a quinolone alkaloid extracted from the fruit of *E. rutaecarpa* [83]. It has been studied for many years, demonstrating functions about anti-proliferation [84], reducing insulin resistance [85], protecting the cardiovascular system [86], and regulating lipid metabolism [87]. In recent years, scholars have gradually begun to apply it to the treatment of AD. Wang et al. performed intragastric administration of intraventricular streptozotocin-induced C57BL/6 mice with Evo for 21 days at 50 mg/kg/day and 100 mg/kg/day [88]. The effect of 100 mg/kg of Evo in mice was similar to that of the positive control group, donepezil-treated mice. Subsequent biochemical tests showed that the administration group significantly reduced the activity of AChE and level of malondialdehyde (MDA) in mice, and the activity of ACh was considered one of the markers of cholinergic function. Because ACh is a neurotransmitter necessary for memory function. MDA is a lipid peroxidation product that increases during oxidative stress. Evo upregulates heme oxygenase 1 (HO-1) by activating Nrf2/ARE pathway to reduce MDA level and inhibit oxidative stress [82]. However, in the later stages of AD, the activity of AChE decreases to 10–15% of the normal values in certain brain regions; however, butyrylcholinesterase (BChE), also known as plasma cholinesterase, remained unchanged or even increased twofold [89]. In fact, we believe that the balance between AChE and BChE inhibition may be more desirable. Studies in vivo have demonstrated that selective BChE inhibitors can increase brain ACh, reduce β-amyloid peptide, and improve cognitive dysfunction [90]. Huang found that Evo combined with a privileged carbamate scaffold has a highly neuroprotective effect on the selectivity and effectiveness of BChE inhibitors, such as the heptyl carbamate of 5-deoxy-3-hydroxynordiamine, which shows a better selection of BChE and AChE [91]. Evo can downregulate the activity of the AKT/GSK-3β signaling pathway and inhibit the activity of nuclear factor NF-κB. As mentioned previously, GSK-3β acts on PI3K and MAPK, ultimately leading to protein deposition. The team of Yuan used SAMP8 and APPswe/PS1ΔE9 transgenic mice with doses of 50 mg/kg/day and 100 mg/kg/day to evaluate the pharmacological efficacy of Evo [92]. The results of the behavioral tests were consistent with those of Wang [88], and higher doses showed better improvement. Western blotting analysis revealed that Evo significantly reduced the accumulation of COX-2 protein, which is one of the important determinants of inflammatory response-mediated cytotoxicity. This inhibition may be achieved by the dephosphorylation of serine/threonine protein kinase B (PKB/Akt) and 70 kDa ribosomal S6 kinase (p70S6k) [83]. Evo can also play a role in anti-inflammation by inhibiting glial activation and neuroinflammatory factors in the hippocampus, including IL-1β, IL-6 and TNF-α. IL-1 induces AChE protein and mRNA expression and increases AChE enzyme activity, which exacerbates cholinergic decline and dysfunction in AD [93]; IL-6 is generally almost undetectable in the adult central nervous system, but it is strongly induced under pathological conditions [94]; TNF-α is a common pro-inflammatory cytokine whose biological effects include stimulating acute-phase reactions and cytotoxicity; COX-2 helps mediate prostaglandins and the production of other inflammatory factors, which itself is regulated by pro-inflammatory mediators [95]. Evo actives IκBα, which is originally used to block the phosphorylation site of NF-κB. Unphosphorylated NF-κB fails to regulate the expression of inflammatory mediators above [96]. Additionally, Ko and his team compared Evo with L-NAME (an NOS inhibitor) and found that Evo can significantly reduce NO production and iNOS protein expression in mouse microglia, likely due to its regulation of interferon IFN-γ-related events [83]. To sum up, Evo can alleviate the pathological symptoms of AD in several ways. First of all, as an alkaloid, the unique nitrogenous base of Evo can more easily bind to AChE one specific site, making it dephosphorylated and reduce its activity, ensuring that ACh can maintain memory function. Secondly, Evo can reduce MDA content and inhibit the adverse effects of oxidative stress by activating Nrf2/ARE pathway. Finally, Evo can reduce neuroinflammation in three ways. (1) It can reduce NO and iNOS in microglia cells by regulating interferon IFN-γ related events; (2) It can be dephosphorylated by serine/threonine protein kinase B and 70 kDa ribosome S6 kinase (p70S6k) to reduce COX-2 protein accumulation; (3) Evo actives IκBα, which induced unphosphorylated NF-κB failing to regulate the expression of inflammatory mediators, including TNF-α, IL-6, iNOS, and COX-2. Therefore, it may reduce the degree of AD CNS dysfunction. These chain reactions confirm that the active ingredients and monomers of TCM have a wide range of pharmacological effects on AD. The pharmacodynamic mechanism of Evo is shown in Fig. 3.

## Berberine

**Fig. 4:**
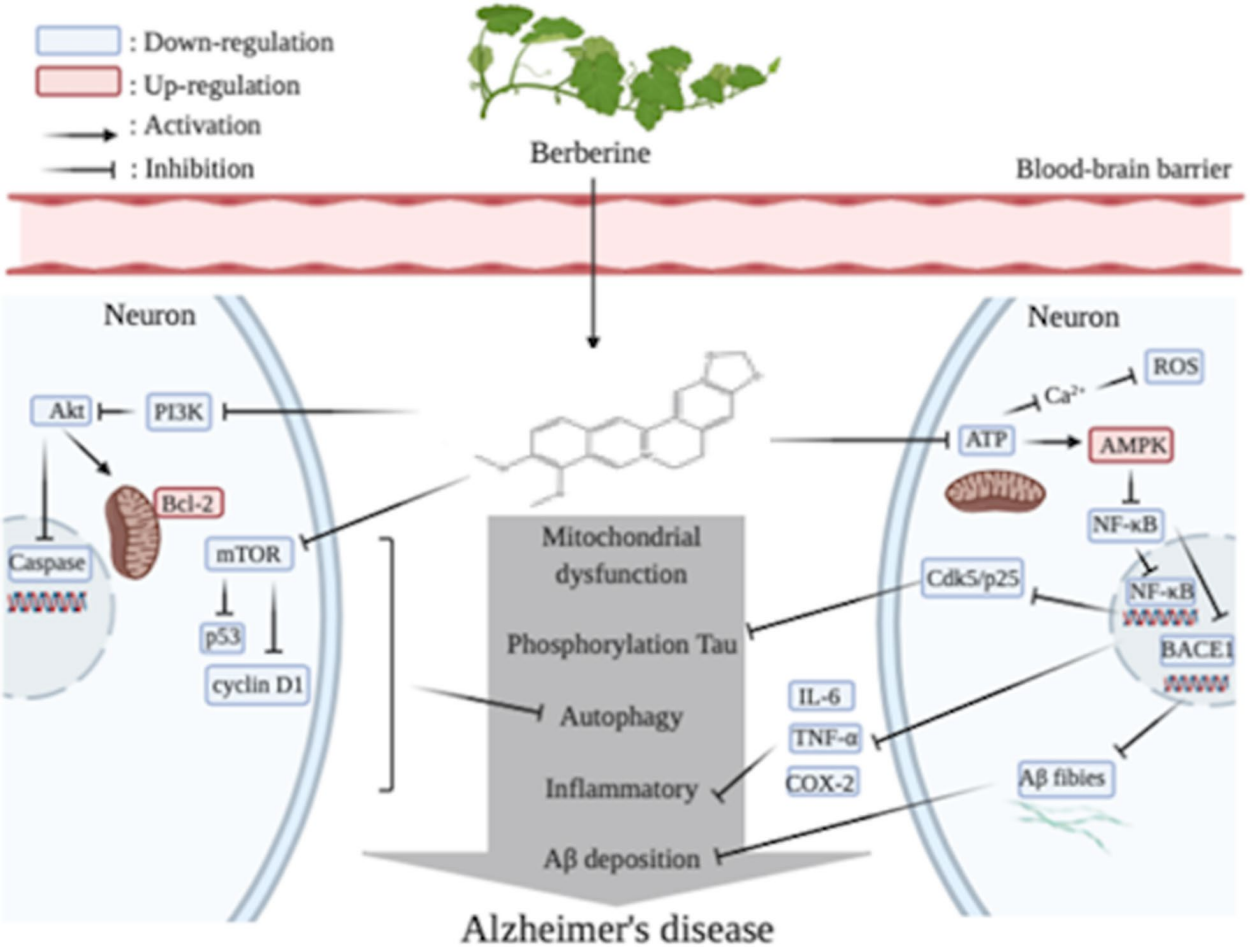
The pharmacodynamic mechanism of BBR for Alzheimer’s disease

Berberine (BBR) is an important alkaloid of *E. rutaecarpa*. According to the data in Table 2, the oral availability of BBR is not as high as that of Evo. However, studies have found that intestinal bacteria can increase the bioavailability of BBR by metabolize it to dihydroberberine. The intestinal absorption rate of dihydroberberine is five times higher than that of BBR. Dihydroberberine is an unstable form that returns to BBR after entering the intestinal wall tissue [97]. BBR can affect the permeability of BBB by upregulating the membrane protein claudin-5 [98]. BBR, as a long-term treatment for OTC drugs, has been safely used clinically in China for decades, and is widely used in health stores in the United States [75]. Numerous studies have shown that BBR can improve symptoms of AD. Hend et al. designed an AD-like model by exposing female adult Sprague–Dawley rats to a mixture of aluminum and cadmium other than fluoride [99]. This mode of modeling was thought to produce neurotoxicity induced by brain ROS, which trigger iron-induced oxidative stress in the CNS and stimulate neuroinflammatory cytokines. Long-term exposure to heavy metals resulted in increased levels of pro-inflammatory molecules such as TNF-α, IL1-β, COX-2 and IL-12 in rat serum and brain compared with those of the blank control group, with IL-6 only in brain tissue. After administration, the levels of pro-inflammatory molecules were significantly decreased. This phenomenon was also the same in cells of other AD models or in animals. The mammalian adenosine monophosphate-activated protein kinase (AMPK) exists as a heterotrimeric complex comprising a catalytic α subunit and regulatory β and γ subunits. AMPK is rapidly activated when the (AMP + ADP)/ATP ratio is increased or in response to an increase in cytoplasmic Ca^2+^ conditions that normally sense cellular stress [100]. AMPK activation downregulates NF-κB activation in various cells and indirectly inhibits NF-κB signaling through its downstream mediators [101], such as Sirtuin 1, an evolutionarily conserved enzyme that mediates the effect of lower eukaryotes on the life extension of caloric restriction [100]. In addition, BBR can downregulate the expression of BACE1 by activating AMPK in mouse neuroblastoma, followed by a decrease in nerve Aβ production in blastoma cells and primary cultured neurons [102]. BACE-1 is the major β-secretase that determines Aβ production. This result is consistent with the animal experiment of Cai [103] in male APP/PS1 transgenic mice. Zhu found that BBR inhibited the expression of BACE by activating the ERK1/2 pathway in HEK293 cells, reducing the production of Aβ40/42 [104]. These results indicate that BBR can also resist AD by lowering Aβ42 and Aβ40 levels. Furthermore, activated NF-κB contributes to Cdk5/p25-induced P-Tau [62, 105]. He et al. found that Tau-6 levels in the hippocampus of BBR-treated AD mice (p-S199, p-S202, p-T205, p-T231, p-S396 and p-S404) significantly decreased, strongly reducing tau hyperphosphorylation in the hippocampus of APP/PS1 mice [106]. Apoptosis and reduction of neurons are also prominent features of AD. Activation of the mTOR signaling pathway mediates aging at the cellular and organism levels [107]. The target of mTOR kinase in mammals is a central inhibitor of autophagy, on which BBR can effect. Luo et al. found that BBR has a protective effect on Aβ-induced cell death in rat cortical neurons by reducing the production of MDA and ROS [108]. Additionally, Akt is a serine/threonine kinase, also known as protein kinase B. Expression of Akt in the CNS is significantly upregulated during cellular stress [109]. In Hu’s study [110], phosphorylation of Akt specifically enhances PI3K’s unique regulation of the p55y promoter activity and reduced cleavage of the pro-apoptotic protein Caspase-3. In sporadic Alzheimer’s-like dementia-type Wistar rats induced by ICV-STZ, the rats treated with BBR for 21 days showed a significant reduction in the number of dead cells [111], but its mechanism that inhibiting apoptosis through downregulating caspase, or Bcl-2, an antagonist that supports apoptosis, needs more studies. BBR has been shown to inhibit the phosphorylation of Bcl-2 in organ-type hippocampal slice cultures in mice against ischemic injury [112]. BBR has also been found to inhibit the expression of cyclin D1 and p53, keeping cells in the G0/G1 phase to prevent neuronal apoptosis [113]. The antioxidant action of BBR has been demonstrated [106, 109, 114] and its mechanism has also been studied. Glutathione (GSH) is an important antioxidant and Glutathione peroxidase (GPx) is a free-radical scavenger. Glutathione synthetase (GSS) induces glutathione levels in the brain. BBR administration increases the levels of GPx-1/2 and GSS in the hippocampus of AD mice, and glutathione reductase converts oxidized GSH into a reduced form, which prevents tissue induced by ROS-induced oxidative stress injury [115]. BBR can induce the nuclear translocation of Nrf2 by activating the AMPK signaling pathway. After nuclear translocation, Nfr2 promotes the transcription and expression of several antioxidant enzymes, increases intracellular SOD, HO-1 and the GSH content, and reduces ROS production and oxidative stress [100]. AMPK also phosphorylates FOXO transcription factors, thereby enhancing the transcriptional activity of FOXO3a, which induces the expression of many antioxidant enzymes and other anti-reverse proteins [116]. To sum up, BBR differs from Evo in that it not only resists oxidation and inflammation, it also resists apoptosis, reduces Aβ and the phosphorylation of tau. All of these functions are performed through AMPK. The mechanism by which BBR exerts AMPK activation may be through mitochondrial targeting. The positioning of BBR in the mitochondria is unstable. Even short-term exposure to ultraviolet light may cause the loss of mitochondrial localization and transfer to the nucleus. The specific target seems to be the respiratory electron transmission chain. Decreasing the ATP content and increasing the AMP/ATP ratio triggered AMPK activation. In addition, BBR significantly increases the activity of AMPK due to excess ROS [100]. Activated AMPK inhibits NF-κB, which is an important node. NF-κB downregulates BACE1 to reduce Aβ, inflammatory factors including TNF-α, IL-6, IL-12, and tau phosphorylation via Cdk5/P25, which could be abnormally activated in AD. Collectively, BBR has various regulatory means for AD, making it a promising multi-effect ingredient for prevention and treatment of AD. The pharmacodynamic mechanism of BBR is shown in Fig. 4.

## Compound-target-pathway network of BBR and Evo

**Fig. 5:**
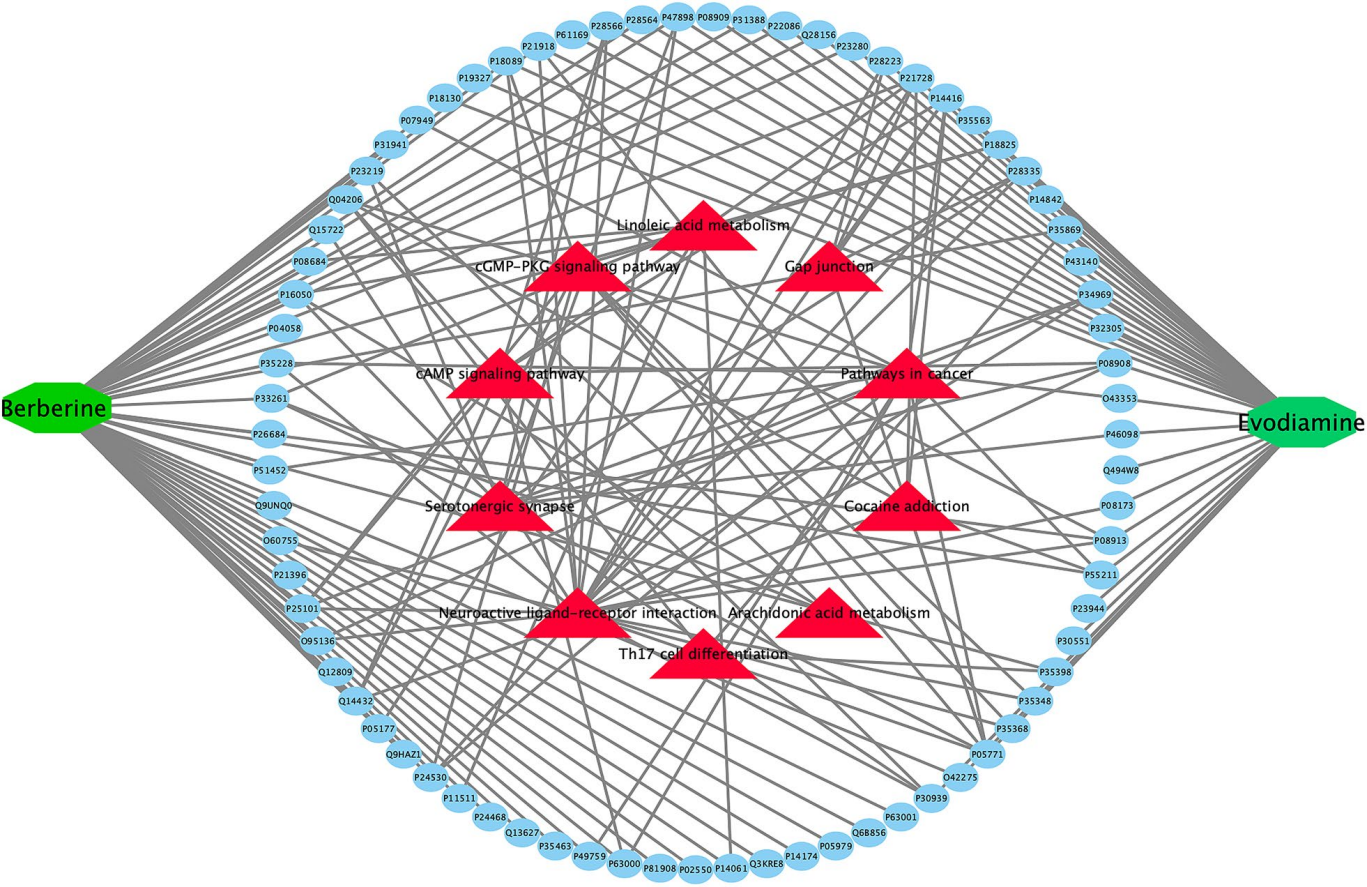
Compound-target-pathways network of BBR and Evo for Alzheimer’s disease

By importing the 3D structures of BBR and Evo into Targetnet, a website that can match the structure of compounds with 623 proteins in the human body, we use proteins with scores greater than 0.5 as the targets of BBR and Evo. In order to better understand the mechanism of action of BBR and Evo on AD, enrichment analysis was performed by DAVID bioinformatics resources, limited to Homo sapiens. Ten KEGG pathways with p-values less than 0.01 were obtained. Pathways include neuroactive ligand-receptor interactions, serotonin synapses and signaling pathways. Herbs contain many compounds, and each compound might target one or multiple targets that involved in different pathways. Therefore, a compound-target-pathway network was further investigated by Cytoscape 3.7 (Fig. 5). Evo and BBR are in the center of the circle. The middle green circle represents the targets of Evo and BBR. The outer red triangles are the relevant pathways obtained by GO enrichment analysis. Through the distribution of interactions, almost all the signal paths with a *p* value less than 0.01 are concentrated at the target of Evo. Each muscarinic receptor subtype has its own unique distribution in the central and peripheral nervous systems. In CNS, muscarine receptors regulate various sensory, cognitive, and motor functions [117, 118]. The network diagram suggests the transmission of some important neurotransmitters, deserves our further attention.

## Discussion and perspective

### Drug metabolism

TCM plays a crucial role in many diseases and at various molecular levels. Multiherb formulas, rather than single herbs, are common in TCM medication. Each herb in a formula has a specific role, including sovereign, minister, assistant, and courier [119]. However, few studies have been conducted on whether there will be toxicity and side effects after mixed administration. In addition, most TCMs acted as precursor drugs, which will present a series of metabolic reactions, including hydrolysis, oxidation and sulfonylation after absorption. And drug responses are often highly variable and greatly influenced by an individual’s ability to metabolize. Therefore, identification and evaluation of efficacy and toxicity of metabolites were quite important for application of TCMs. TCM is generally taken by oral administration, but the target of the disease is distributed throughout the whole body, especially for the brain disease of AD. The distribution of TCMs after administration, such as whether they can pass through the BBB, is worthy to study. Fortunately, nano-drug delivery systems have become a research hotspot in the field of drug delivery in recent years [120]. Nano-carriers can be designed into different sizes, shapes and surface charges, and their penetration and targeting abilities can be enhanced through modification. Therefore, the nano-drug delivery system has become the most promising means to reach the target site through the BBB for the treatment of AD.

### Experimental animal model

The commonly used animal models for AD are mainly divided into transgenic and non-transgenic models. The former models include APP transgenic mice, tau transgenic mice, PS transgenic mice and APP/PS-1 double transgenic mice. The other one includes models induced by Aβ, cholinergic system damage, metabolic disorders. In recent years, great progress has been made in the study of animal models of AD treated with TCM. However, animal models currently known can only simulate the main pathological, neurobiochemical and partial behavioral changes of AD, and none can fully simulate the performance of human memory loss and dementia. Additionally, human sex differences cannot be replicated in mouse models. Because there are many differences among models, the same drug must be tested in different models to assess the reproducibility of efficacy and safety [121, 122]. To understand the higher cognitive function in the human brain and how the brain dysfunction in AD progression, experimental animals that are more similar to humans are essential. One way to overcome this key obstacle is to generate a viable AD model in non-human primates (NHPs). Because human and NHP brains have considerable similarities in the overall structure and tissue structure of functional networks [123]. Although NHPs and rodents are the best animal options for AD modeling, the high cost and scarce resources limit their extensive use. However, the main challenge for the drug development of AD is the lack of a clear understanding of the pathogenesis and pathophysiology of AD. For example, a growing number of studies have suggested a link between AD and inflammation. Unfortunately, without appropriate animal models, the relationship between inflammatory process stages and AD progression remains poorly clarified. This could explain, at least in part, the failure of clinical trials using anti-inflammatory molecules whose efficacy has been significantly demonstrated in preclinical investigations [17]. Therefore, it is inadequate to support the use of new drugs to prevent and treat AD based on the current evidence alone. This uncertainty is mainly due to the limitations of research methods, such as a poor animal model, a relatively small sample size, improper measurement methods and invalid statistical analysis [124].

### Experimental period

AD is a progressive chronic disease, and experiments that verify the potential value of TCM in the prevention and treatment of AD only focus on the change of symptoms and have a short treatment cycle (< 6 months), both of which are quite inadequate. Additionally, the current drug design of AD has shifted from a single-targeted approach (mainly centered on amyloid) to the multi-target one, and from treatment at the later stage of disease to the prevention strategy at the early stage of development. Shi et al. conducted experiments on AD patients treated with conventional therapy and conventional therapy combined with herbal therapy (CT + H) in a clinical setting [125]. MMSE was performed every 3 months for cognitive function, and the longest follow up was 24 months. CT + H showed significant improvement in AD patients compared with CT alone. The cognitive function of moderate patients decreased significantly and that of mild patients was basically stable for more than 2 years. Certainly, the individual heterogeneity of patients should be considered, as well as the different physiological and biochemical changes in the stage of disease and other conditions, to correctly implement TCM for the prevention and treatment of AD. Undoubtedly, the reasonable experimental period regardless of clinical and animal experiments should be taken as an important factor in the study of TCM for chronic disease.

## Conclusions

TCM has a long history of treating neurodegenerative diseases, and the composition and mechanism of some TCMs have gradually been proven. This review summarized several pathogenic mechanisms and potential biomarkers of AD, as well as the pharmacological efficacy and mechanism of action of berberine and evodiamine from *E. rutaecarpa* for AD. Additionally, the limitations of current studies on AD and TCM were also discussed, the drug metabolism, model animal and experimental period should be considered. The review lays a foundation for the subsequent expansion of therapeutic targets of AD and could promote the development of effective disease-modifying TCM monomers or extracts.

## Data Availability

All the data used to support the findings of this study are available from the corresponding author upon reasonable request.

## Abbreviations

ACh: Acetyl choline
AChE: Acetylcholinesterase
AD: Alzheimer’s disease
AMPK: Adenosine monophosphate-activated protein kinase
ATN: Amyloid deposition (A), pathology tau (T) and neurodegenerative changes (N)
BACE 1: β-Amyloid precursor protein lyase 1
BBB: Blood–brain barrier
BBR: Berberine
BChE: Butyrylcholinesterase
CNS: Central nervous system
CSF: Cerebrospinal fluid
CT + H: Conventional therapy combined with herbal therapy
DL: Drug-likeness
Evo: Evodiamine
FDA: Food and Drug Administration
GSH: Glutathione
GSK-3β: Glycogen synthase kinase 3β
HO-1: Heme oxygenase 1
MAPK: Proliferating protein kinase
MDA: Malondialdehyde
NFκB: Nuclear factor κ-light-chain-enhancer of activated B-cell
NHPs: Non-human primates
NMDA: *N*-methyl-d-aspartic acid
NTP: Neuronal thread protein
OB: Oral bioavailability
PET: Positron emission tomography
PI3K: Phosphoinositide 3-kinases
P-Tau: Phosphorylated-Tau
PTP: Pancreatic thread protein
TCM: Traditional Chinese medicine
TCMSP: Traditional Chinese medicine systems pharmacology database and analysis platform
TNF: Tumor necrosis factor
